# Metabolic reprogramming in hepatocellular carcinoma: an integrated omics study of lipid pathways and their diagnostic potential

**DOI:** 10.1186/s12967-025-06698-7

**Published:** 2025-06-11

**Authors:** Peng Dai, Jing Feng, Yanyan Dong, Shujing Zhang, Jianghong Cao, Xiaopeng Cui, Xueliang Qin, Shiming Yang, Daguang Fan

**Affiliations:** 1 Department of Hepato-Pancreatic-Biliary Surgery, Shanxi Provincial People’s Hospital, Taiyuan, 030000 China; 2 Department of Gastroenterology, Shanxi Provincial People’s Hospital, Taiyuan, 030000 China; 3 Department of Pathology, Shanxi Provincial People’s Hospital, Taiyuan, 030000 China; 4 Department of Digestive Endoscopy, Shanxi Provincial People’s Hospital, Taiyuan, 030000 China; 5 Department of Medical Intensive Care Unit, Shanxi Provincial People’s Hospital, Taiyuan, 030000 China

**Keywords:** Hepatocellular carcinoma (HCC), Lipid metabolism, Metabolic reprogramming, Multi-omics analysis, Diagnostic biomarkers

## Abstract

Metabolic reprogramming is an important cancer hallmark. Recent studies have indicated that lipid metabolic reprogramming play a potential role in the development of hepatocellular carcinoma (HCC). However, the underlying mechanisms remain incompletely understood. In this study, we employed an integrated multi-omics approach, combining transcriptomic, proteomic, and metabolomic analyses, to explore the lipid metabolism pathways in HCC and evaluate their diagnostic potential.

We collected ten pairs of HCC tissues (HCT) and adjacent non-tumor tissues (ANT) from patients undergoing surgical resection. Transcriptomic analysis identified 4,023 differentially expressed genes (DEGs) between HCT and ANT, with significant enrichment in lipid metabolism-related pathways, including fatty acid degradation and steroid hormone biosynthesis. Proteomic analysis revealed 2,531 differentially expressed proteins (DEPs), further highlighting lipid metabolism as a critical driver of HCC development. Metabolomic profiling identified 88 differentially expressed metabolites (DEMs), with notable alterations in lipid-related metabolites. Integrated analysis of transcriptomic, proteomic, and metabolomic data identified six key genes (LCAT, PEMT, ACSL1, GPD1, ACSL4, and LPCAT1) involved in lipid metabolism, which exhibited significant changes at both mRNA and protein levels and correlated strongly with lipid-related metabolites in HCT. Additionally, nine lipid-related metabolites were identified as potential diagnostic biomarkers for HCC, with six metabolites demonstrating high discriminative ability (AUC > 0.8) between HCT and ANT.

Our findings provide new insights into the molecular mechanisms of lipid metabolism reprogramming in HCC, emphasize the critical role of lipid metabolism in its pathogenesis. The identification of lipid-related metabolites as potential diagnostic biomarkers holds significant promise for early detection and improved clinical management of HCC. The integrated multi-omics approach as a powerful tool for identifying novel biomarkers and therapeutic targets.

**Supplementary Information:**

The online version contains supplementary material available at 10.1186/s12967-025-06698-7.

## Introduction

Primary liver cancer is one of the most prevalent cancers worldwide, with a mortality rate that ranks third among all malignant tumors. Hepatocellular carcinoma (HCC) is the most significant type of primary liver cancer [1]. Although recent progress in early diagnosis and treatment has enhanced the short-term prognosis of patients with HCC, the long-term prognosis remains unsatisfactory even after curative treatment [2, 3]. However, HCC is characterized by early metastasis, rapid progression, and a lack of effective early diagnostic markers. These characteristics render its diagnosis exceedingly challenging. Consequently, HCC is frequently detected at an advanced stage, when treatment options are limited and the prognosis is poor. Furthermore, the absence of early diagnostic markers further complicates HCC diagnosis [3, 4]. Therefore, it is of great significance to find a biomarker suitable for early diagnosis ofHCC to prevent liver cancer and prolong the life expectancy of patients.

Metabolic reprogramming, a significant hallmark of cancer, has emerged as a focal point of research in oncology [5]. Due to the requirements of rapid cell proliferation and metastasis of cancer cells, they often face hypoxic and hypo-nutrient conditions. Moreover, they must undergo adaptive bioenergetic changes to meet high energy demands or create a favorable microenvironment for their growth. This process is termed “metabolic reprogramming”, which results in enhanced catabolic metabolism and anabolic metabolis in cancerous tissues compared with normal tissues [5, 6]. Metabolites arising from metabolic reprogramming also exert cancer-promoting functions by modulating signaling pathways, epigenetic states, and cellular differentiation [7], Therefore, a substantial amount of research has also emphasized the utilization of metabolites for cancer diagnosis [8, 9]. The most extensively studied metabolic alteration is the Warburg effect [10]. Another metabolomic aberration that has been subject to in-depth analysis pertains to the augmented glutamine metabolism [11]. Additionally, notwithstanding the comparatively diminished attention accorded to the perturbations in lipid metabolic metabolism among cancer cells, emerging evidence highlights the crucial role of lipid metabolic reprogramming in carcinogenesis [12]. Beyond the established alterations in glucose and glutamine metabolism, dysregulated lipid metabolism has been increasingly recognized as a key driver of HCC progression. Aberrant lipid homeostasis-characterized by altered fatty acid synthesis, degradation, and storage-not only provides the energy and biosynthetic precursors for rapid tumor proliferation but also modulates membrane composition and signaling pathways, thereby promoting invasion, metastasis, and drug resistance [13]. Mitochondrial dysfunction further exacerbates lipid accumulation, activating the mTOR pathway and promoting tumorigenesis [14]. Moreover, changes in lipid composition are associated with resistance to targeted therapies, as modifications in lipid-modifying enzyme activity help cancer cells evade drug-induced cell death [15]. Integrated multi-omics analyses have identified distinct lipidomic signatures, including elevated monounsaturated phosphatidylcholines, which correlate with increased cell proliferation and poorer clinical outcomes [16]. In chronic liver diseases such as nonalcoholic fatty liver disease (NAFLD) and viral hepatitis, dynamic alterations in the hepatic lipidome not only exacerbate liver injury but also elevate the risk of HCC development [17]. Furthermore, targeting lipid metabolic pathways—for instance, through modulation of the AMPK-ACC-PPARα axis—has shown potential in reducing hepatic steatosis and slowing the progression to HCC [18]. Despite these advances, the molecular mechanisms underlying lipid metabolic reprogramming in HCC remain incompletely understood. Further exploration of these mechanisms is crucial for elucidating tumor pathogenesis and identifying novel therapeutic targets.

Multi-omics analysis technologies are often utilized to explore the pathogenesis associated with cancer. The comprehensive analysis of different types of omics data can provide perspectives into the pathogenesis of cancer [19, 20]. In addition, multi-omics analyses are frequently used to elucidate potential global changes in research objects and screen candidate molecules for further studies [21]. In this study, a comprehensive analysis of transcriptomics, proteomics and metabolomics was performed to explore the mechanisms underlying the lipid metabolic reprogramming in HCC, through multi-omics analysis, we seek to elucidate the regulatory mechanisms underlying lipid metabolic reprogramming in HCC through the discovered key genes, proteins and pathways, providing guidance for further research on the pathogenesis of hepatocytes.

## Materials and methods

### Patient recruitment and tissues sample collection

A total of ten pairs of HCC tissues (HCT) and adjacent normal tissues (ANT) were collected from the patients who underwent resections without preoperative therapy in the Shanxi Provincial People’s Hospital (Taiyuan, China) from April 2021 to May 2023. The diagnosis of HCC was confirmed by postsurgery pathology. Cancerous tumors and adjacent tissues were cut into 3–5 pieces of ~ 0.5 cm diameter stored in liquid nitrogen. The clinical pathologic traits of patients including gender, age, tissue collection time, and the largest diameter of the tumor are summarized in Table 1. This study was performed following the Helsinki Declaration and approved by the clinical research ethics committee of the (SXSPH-2021–EC-018). Written informed consent was obtained from each patient according to the policies of the committee.

**Table 1:** Clinical characteristics of 10 HCC patients

Features	Patient cohort (*n* = 10)
Age (mean ± SD)	57.94 ± 6.72
Gender (male%)	9 (90%)
BMI (kg/m2)	21.38 ± 2.57
**Tumor size**	
< 5 cm	3(30%)
>=5 cm	7(70%)
**Grade**	
G1-G2	6 (60%)
> G2	4 (40%)

### Histopathological analysis

To evaluate the histological architecture, hematoxylin and eosin (H&E) staining was applied to the representative tissue slices of HCT and ANT. The H&E staining was carried out using a Hematoxylin and Eosin Staining Kit (Beyotime, C0105M, Shanghai, China). Subsequently, the stained slices were examined under a light microscope, and images were captured for the purpose of further analysis.

### Transcriptomic analysis

Total RNA was extracted from the HCT and ANT using Trizol reagent (Invitrogen, Carlsbad, CA, USA) according to the manufacturer’s instructions. Then, the RNA quantity and quality were checked by multiple techniques, Including NanoDrop 2000 to measure concentration and purity of total RNA, 1% agarose gel electrophoresis for visual inspection of RNA bands, Qubit^®^ 4.0 Fluorometry for precise quantification. The cDNA libraries were generated using the NEBNext^®^ UltraTM Directional RNA Library Prep Kit for Illumina following the manufacturer’s recommendations.

The sequencing of the samples was done on the Illumina HiSeq 6000 using the PE150 sequencing strategy. The quality control of the clean reads was done using the Trimmomatic v0.33 with the default parameters and then clean reads were mapped to the human genome (Homo_sapiens.GRCh38) using hisat2 v2.1.0 with the following parameters: --dta-cufflinks --score-min L,0,-0.6 [22]. Additionally, the mapped reads for each gene were counted by HTseq v2.0.9 [23]. Gene expression level quantification was evaluated based on fragments per kilobase of transcript per million fragments mapped (FPKM). Principal component analysis (PCA) was performed to compare the FPKM values of the expressed gene profiles among HCT and ANT using the PRCOMP function in R. Differential expressed genes (DEGs) were identified by DESeq2 based on log2Fold Change > 0.584 with *p*-value < 0.05 [24]. Gene Ontology (GO) and Kyoto Encyclopedia of Genes and Genomes (KEGG) enrichment analyses were performed using the R package cluster Profiler v4.0 and ggplot2 [25]. All genes were taken into gene set enrichment analysis (GSEA) analysis by GSEA function of R packages clusterProfiler(v3.18.0) and and GseaVis v0.0.5 [26]. Gene sets of which *p*-value < 0.01 and FDR < 0.05 were regarded as significantly enriched term.

### Proteomic analysis

HCT and ANT samples were homogenized three times in the ice-cold lysis buffer (8 M urea, 1% Protease Inhibitor Cocktail) using a tissue lyser (60 Hz, 2 min). then The homogenate was centrifuged for 14,000 rpm 30 min in 4 °C, and the supernatant was collected. The protein content of the supernatant was determined using the BCA Protein Assay Kit (Beyotime Biotechnology, Shanghai, China). Then, 50 g proteins were suspended in 50µL of solution, reduced with 1 M dithiothreitol (DTT) at 55 °C for 1 h, and alkylated with 5 µL 1 M iodoacetamide in the dark for 1 h. The material was then precipitated using 300 µL of prechilled acetone. Trypsin (Promega) was used to digest the precipitate overnight.

Next, we performed high PH reversed phase separation. The peptide mixture was redissolved and fractionated by high pH separation utilizing an Ultimate 3000 system (ThermoFisher Scientific, MA, USA) linked to a reverse phase column (XBridge C18 column, 4.6 mm × 250 mm, 5 m) (Waters Corporation, MA, USA). A linear gradient was used to produce high pH separation, ranging from 5 to 45% solvent B( 97% ACN, pH 9.8) in 40 min. The elution peaks were monitored intently and fractions were collected every minute. Guided by peak chromatograms, the fractions were combined judiciously, resulting in ten fractions that were immediately freeze-dried for sample preservation and subsequent utilization.

Peptides were redissolved in 30 µL solvent A (0.1% formic acid in water) and analyzed on an Orbitrap Fusion Lumos linked to an EASY-nLC 1200 system using on-line nanospray LC-MS/MS (Thermo Fisher Scientific, MA, USA). The mass spectrometer was set to the data-dependent acquisition(DDA) mode and automatically transitioned between MS and MS/MS modes. Raw DDA data were processed and analyzed using Spectronaut X (Biognosys AG, Switzerland) with default parameters to build an initial target list. The q-value (FDR) limit was set at 1% on the precursor and protein levels. Pulsar was then used to create a database from the DDA acquisition mode data. The DIA data were compared to the DDA reference database to identify proteins. The raw protein intensity will be normalized by method “medium”, Hierarchical clustering was performed using pheatmap package. Principal component analysis (PCA) was performed using metaX package. T test was used for statistical differential analysis and a cut of *p*-value < 0.05 and log2fold change ≥ 0.584 was used to select statistically differential expressed proteins (DEPs). Hypergeometric-based enrichment analysis with KEGG and Wiki Pathway, Gene Ontology and Reactome Pathway were performed to annotate protein sequences.

### Metabolomic analysis

The collected HCT and ANT samples were thawed on ice and then mixed with 300 µL of precooled acetonitrile and 200 mg of ceramic beads. Subsequently, the resulting mixture was homogenized and centrifuged at 4 °C and 12,000 rpm for 10 min. Then, the supernatant was centrifuged again under the same conditions (4 °C, 12,000 rpm for 10 min) and then filtered through 0.22 μm syringe filters prior to analysis. In addition, a quality control (QC) sample was also prepared, which is a mixture of all the samples for signal correction.

The metabolites were introduced into the LC-MS/MS system for analysis. The LC-MS/MS analyses were carried out by means of a Vanquish UHPLC system, and were coupled with an Orbitrap Q Exactive series mass spectrometer (Thermo Fisher Scientific, MA, USA).

The preprocessed HCT metabolomic data were separated from the ANT metabolomic data. The metabolites were quantified using a triple quadrupole mass spectrometry system in the multiple reaction monitoring mode and then identified using the MetWare database and other publicly available metabolite databases, including HMDB, KEGG, METLIN and others based on their mass-to-charge ratio (m/z), retention time, and fragmentation patterns. The Analyst (v1.6.1) software (AB SCIEX, ON, Canada) was used to process the mass spectrometry data. After obtaining the mass spectra of the metabolites in different samples, the peak areas of all mass spectral peaks were integrated and the mass spectral peaks of the same metabolite in different samples were integrated and corrected. Furthermore, a principal component analysis (PCA) was performed to analyze the metabolite data. A orthogonal partial least-squares-discriminant analysis (OPLS-DA) was completed to maximize the differences in metabolites between groups and identify the metabolites. The variable importance in projection (VIP) value of the OPLS-DA model was combined with the *p*-value or fold-change data in the univariate analysis to screen for differentially expressed metabolites (DEMs), Metabolites with a VIP ≥ 1 and a fold-change ≥ 2 or ≤ 0.5 were considered to be significantly different. Hierarchical clustering and correlation analysis were conducted to accurately screen differential metabolites and measure their correlations among those that were significantly differentially expressed. Subsequently, the KEGG and MetaboAnalyst databases were employed to elucidate the underlying metabolic pathways. Additionally, receiver operating characteristic (ROC) curve analysis and correlation analysis were employed to evaluate the potential of the differentially expressed metabolites related to lipid metabolic as diagnostic biomarkers for HCC.

### Integrated analysis of transcriptomics, proteomic, and metabolomics

To elucidate the correlation between DEGs and DEPs in the HCT and ANT groups, we employed a Venn diagram analysis to identify overlapping genes, The analysis was conducted using R software package. The data of the identified DEGs consistent with DEPs were subsequently subjected to the Kyoto Encyclopedia of Genes and Genomes (KEGG) pathway analysis to explore the potential functions. Additionally, the Pearson correlation analysis was employed to evaluate the DEMs related to lipid metabolic and the genes that most significant alterations in both RNA and protein levels by R package corrplot (v0.95). Only correlations with *p*-values ≤ 0.05 and absolute Pearson coefficients|R-value|≥ 0.5 were deemed statistically significant. Based on these significant Pearson correlation coefficients, gene-protein-metabolite co-regulatory networks were constructed using Cytoscape (version 3.7.1, Seattle, WA, USA). Network topology parameters, including the average number of neighbors and network density, were subsequently calculated to assess the overall connectivity and complexity of the network.

### Expression validation

To confirm the sequencing results, we selected six genes (LCAT, PEMT, ACSL1, GPD1, LPCAT1, ACSL4) from the identified DEGs consistent with DEPs for validation, and specific primers for qRT-PCR analysis were designed using Premier 5 Designer software and the primers were controlled by the BLAST tool against nr database (NCBI). the primer sequences are given in Table 2. qRT-PCR was performed using a ABI ViiA 7 Real-Time PCR System (Applied Biosystems, Foster City, CA) and SYBR Green PCR Master Mix (TaKaRa BioTechnology, Kusatsu, Japan). The reaction mix included 7.5µL of 2x Universal Blue SYBR Green PCR Master Mix, 1.5µL of F/R Primers (2.5µM), 2.0µL of cDNA, and 4µL of nuclease-free water. GAPDH was used as the endogenous control and all reactions were performed in triplicate. Relative gene expression was calculated using the comparative cycle threshold (2 ^−△△CT^ ) method.

**Table 2:** Primer sequence

genes	Forward Sequence (5′→3′)	Reverse Sequence(5′→3′)
LACT	CAGTCCTGGAAGGACCACTTCA	GAAGTCGTGGTTATGCGCTGCT
PEMT	GGAGGTGGTGGAAGTGTGG	GGTGGTGGTGGTGGTGGT
ACSL1	ATCAGGCTGCTTATGGACGACC	CCAACAGCCATCGCTTCAAGGA
GPD1	GCCACTACTGTGCCTTTGAGTC	CCCTCAGAGAATCGCCAGTACT
LPCAT1	CGACCTATTCCGAGCCATTGAC	GTGAGGTCTCTGCACAGCTTTC
ACSL4	CCTTTGGCTCATGTGCTGGAAC	GCCATAAGTGTGGGTTTCAGTAC
GAPDH	TGTGTCCGTCGTGGATCTGA	TTGCTGTTGAAGTCGCAGGAG

The proteins corresponding to the same five genes were verified through Western Blotting, and the specific operations are as follows: Total protein from tissue samples was extracted in 1*SDS buffer and the protein concentrations were calculated with a Pierce BCA protein assay kit (Pierce, Rockford, IL). Equivalent quantities of proteins were separated by 15% SDS–PAGE gel and transferred to PVDF membranes (Millipore), following blocked with 5% non-fat milk, the membranes then incubated at 4 °C overnight with primary antibodies (LCAT, PEMT, GPD1, LPCAT1, ACSL4 and GAPDH, Santa ), and washed three times with Tris-buffered saline tween(TBST), then incubated with the secondary corresponding goat anti-mouse horseradish peroxidase (HRP)-conjugated antibody at room temperature for 2 h, Subsequently, washing, and imaging. The protein levels were normalized to those of GAPDH.

### Statistical analysis

Statistical analyses were performed using SPSS (version 26) and the R software (version 4.0). Comparisons between two groups were calculated by Student’s t test. In addition, spearman correlation analysis was used to calculate the relationship between two groups based on relative abundance. *p*-value < 0.05 was considered statistically significant.

## Results

### Patient characteristics

Table 1 presents the characteristics of the patient cohort, a total of 10 patients were enrolled in this study. The mean age of the cohort was 57.94 ± 6.72 years, indicating a predominantly middle-aged population. The majority of the patients were male (9/10, 90%), while one patient was female (1/10, 10%). The mean body mass index (BMI) was 21.38 kg/m^2^, with a standard deviation of ± 2.57 kg/m^2^, suggesting a relatively normal weight distribution based on the World Health Organization (WHO) classification criteria. Tumor characteristics were assessed and stratified based on size and histological grade. In terms of tumor size, 30% of the patients (3/10) had tumors < 5 cm in diameter, whereas 70% (7/10) presented with tumors ≥ 5 cm. Tumor grade was classified into two categories: low to intermediate grade (G1-G2), observed in 60% of patients (6/10), and high grade (> G2), observed in 40% of patients (4/10).

The heterogeneity observed within the patient cohort, particularly with respect to tumor burden and histological aggressiveness, may significantly influence subsequent clinical outcomes. The high prevalence of larger tumors (70% of patients with tumors ≥ 5 cm) may indicate a tendency towards delayed diagnosis or advanced disease presentation within this cohort. Furthermore, the observed BMI values, which are slightly lower than the general population average, could potentially suggest the presence of disease-related cachexia or other factors specific to this patient population. These findings provide crucial context for the interpretation of clinical and pathological data within the study.

### Histopathological observation of HCT and ANT

**Fig. 1:**
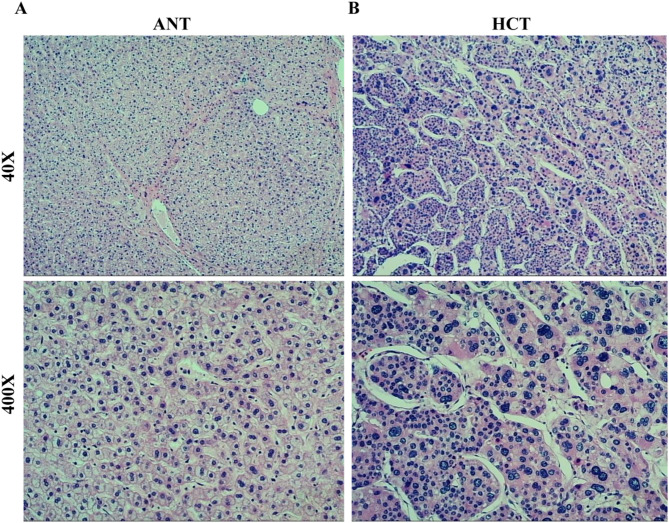
H&E staining of HCT and ANT: (**A**) HCT showing disorganized architecture, cellular pleomorphism, atypical nuclei, and mitotic figures. (**B**) ANT with preserved lobular structure and uniform hepatocytes

Hematoxylin and eosin (H&E) staining of the ANT delineates a well-organized hepatic lobular architecture, with hepatocytes radially arrayed around a central vein. Hepatocytes exhibit a polygonal morphology, featuring round nuclei with finely granular chromatin and prominently visible nucleoli (Fig. 1A). In stark contrast, HCT is characterized by a disorganized cellular architecture, with neoplastic cells displaying marked pleomorphism. The nuclei are enlarged, hyperchromatic, and irregularly shaped, with pronounced nucleoli. The cytoplasm of these cells appears eosinophilic or exhibits vacuolation. Moreover, the tumor tissue is interspersed with areas of fibrosis and evidence of vascular proliferation (Fig. 1B).

### Transcriptomic analysis between HCT and ANT

**Fig. 2:**
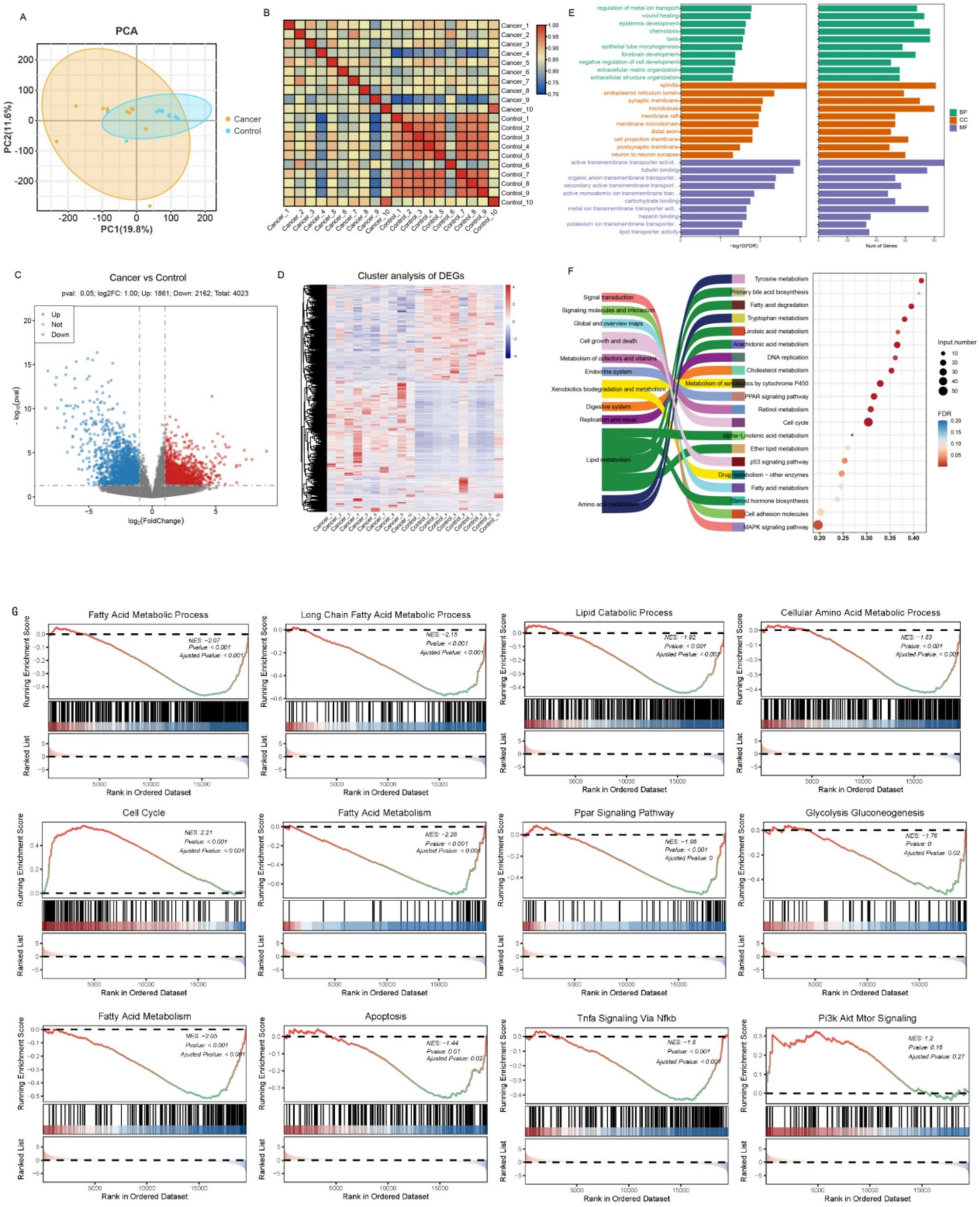
Transcriptomic analysis of ANT and HCT: (**A**) Principal component analysis (PCA) scatter plots of transcriptomes between tissue samples. (**B**) Heatmap of correlation between samples. (**C**) Volcano plot depicting the differentially expressed genes (DEGs) in HCT and ANT. The x-axis represents the log2-fold change in gene expression between the different groups. The y-axis represents the significance level of the expression difference, The red and blue dots represent significantly up-regulated and down-regulated DEGs, respectively. (**D**) Heatmap visualizing DEGs between HCT and ANT based on hierarchical clustering analysis. (**E**-**F**) Gene Ontology (GO) and KEGG Pathway Enrichment Analysis of DEGs. (**G**) GSEA analysis showed key biological processes and pathways involved in DEGs

High-throughput sequencing produced 962,664,954 reads, totaling 144.3Gb of data. After filtering, a total of 958,869,566 clean reads of 150 bp length were obtained, representing 143.84Gb, with a Q20 value of 97.47% and a GC content of 48.21%, demonstrating high sequencing quality of these reads, exceeded 98% could be mapped human genome, indicating a high utilization rate of reads (Supplementary Table S1). Meanwhile, Principal component analysis (PCA), combined with correlation analysis between samples, revealed good repeatability within groups HCT and ANT, with significant differences between the groups (Fig. 2A and B ). The above mentioned results indicated that our experiment was reproducible and reliable, which could be used for further analysis.

The gene expression patterns between groups HCT and ANT were visualized using heatmaps in (Fig. 2D ), The analysis revealed 4023 differentially expressed genes (DEGs) in the HCT group compared to the ANT group, including 1861 upregulated and 2162 downregulated (Fig. 2C, Table S2). To better understand the biological functions of the DEGs, the DEGs were evaluated by GO analysis, KEGG pathway analysis, and Gene Set Enrichment Analysis (GSEA).

GO enrichment analysis showed that DEGs were clustered into three parts: biological process (BP), cellular component (CC), and molecular function (MF). In the BP group, DEGs were mainly enriched in the negative regulation of cell development and epidermis development. In the MF group, DEGs were mainly enriched in active transmembrane transporter activity, organic anion transmembrane transporter, secondary active transmembrane transport, active monoatomic ion transmembrane transport and tubulin binding. For CC group, the DEGs were enriched in spindle (Fig. 2E, Table S3).

KEGG pathway enrichment analysis indicated that the DEGs were significantly enriched in multiple metabolic and signaling pathways, including primary bile acid biosynthesis, fatty acid degradation, linoleic acid metabolism, hormone biosynthesis, fatty acid metabolism, biosynthesis of unsaturated fatty acids, alpha-Linolenic acid metabolism, Enter lipid metabolism, Steroid hormone biosynthesis and PPAR signaling pathway (Fig. 2F, Table S4), These findings suggest that lipid metabolism-related pathways play a key role in the occurrence and progression of HCC.

Gene set enrichment analysis (GSEA) further demonstrated that cell cycle and PI3k Akt Mtor Signaling pathways were significantly upregulated in HCT compared to ANT. Conversely, several lipid-associated metabolic pathways, including the Fatty Acid Metabolic Process, Long Chain Fatty Acid Metabolic Process, Lipid Catabolic Proces, Fatty Acid Metabolism, as well as other pathways such as Cellular Amino Acid Metabolic Process, Ppar Signaling Pathway, Glycolysis Gluconeogenesis, Apoptosis, and Tnfa Signaling Via Nfkb were significantly downregulated in HCT (Fig. 2G). These findings suggest significant alterations in lipid-related metabolic pathways, indicating a metabolic reprogramming in HCC involving lipids metabolism.

### Proteomic analysis between HCT and ANT

**Fig. 3:**
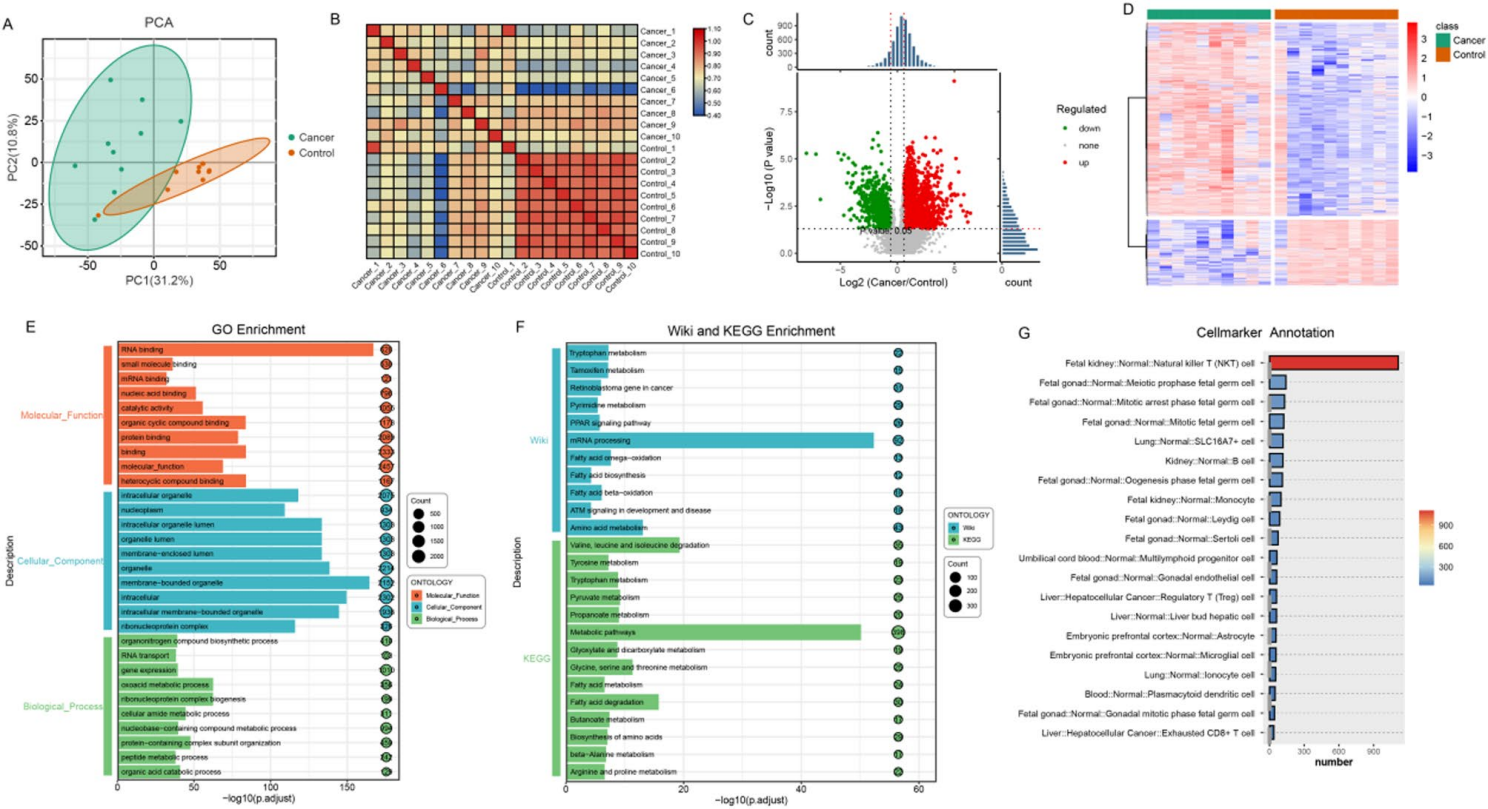
Proteomic analysis of ANT and HCT: (**A**) Principal component analysis (PCA) scatter plots of proteomics between tissue samples. (**B**) Heatmap of correlation between samples. (**C**) Volcano plot depicting the differentially expressed proteins (DEPs) in HCT and ANT. The x-axis represents the log2-fold change in protein expression between the different groups. The y-axis represents the significance level of the expression difference, The red and green dots represent significantly up-regulated and down-regulated DEPs, respectively. (**D**) Heatmap visualizing DEPs between HCT and ANT based on hierarchical clustering analysis. (**E**-**F**) Gene Ontology (GO), KEGG and Wiki Pathway Enrichment Analysis of DEPs. (**G**) Protein Counts in Different Tissues and Cell Types

We also characterized the proteomic of the HCT and ANT groups (Table S5). Consistent with the transcriptomic findings, LC-MS/MS analysis confirmed that the proteome of HCT was significantly altered compared to the ANT group. Principal component analysis (PCA) combined with correlation analysis was performed to assess the relationships in protein expression between the HCT and ANT groups. As expected, the proteomic profiles of the HCT samples were clearly separated from the ANT samples, with greater dispersion observed in the HCT group. These results indicated a high degree of correlation within each group and clear separation between the groups (Fig. 3A and B).

Additionally, protein expression patterns between the HCT and ANT groups were visualized using heatmaps (Fig. 3D). A total of 2,531 differentially expressed proteins (DEPs) were identified, including 641 downregulated and 1,890 upregulated proteins (Fig. 3C, Table S6). Following the identification of the DEPs, Gene Ontology (GO), Wiki Pathways, and KEGG pathway enrichment analyses were conducted to further explore their biological functions.

GO enrichment analysis revealed the following results, In the Biological Process (BP) category, DEPs were predominantly enriched in gene expression and oxoacid metabolic process. Regarding the Cellular Component (CC) aspect, enrichment was mainly observed in terms related to intracellular structures, membrane-bounded organelle, and intracellular organelle. The Molecular Function (MF) analysis, DEPs were significantly enriched in RNA binding and heterocyclic compound binding (Fig. 3E, Table S7). KEGG pathway enrichment analysis revealed significant enrichment in 14 pathways, with the metabolic pathway being notably prominent. Wiki enrichment analysis further identified significant enrichment of DEPs in mRNA processing, fatty acid omega-oxidation, and amino acid metabolism (Fig. 3F, Table S8). The enrichment analysis of DEPs within the context of diverse tissues and cellular compartments demonstrated a pronounced and statistically significant enrichment in natural killer (NK) cells. This observation is characterized by NK cells exhibiting the highest relative abundance of differentially regulated proteins, suggesting their integral involvement in the pathophysiological mechanisms associated with the tumorigenesis (Fig. 3G).

### Metabolomic analysis between HCT and ANT

**Fig. 4:**
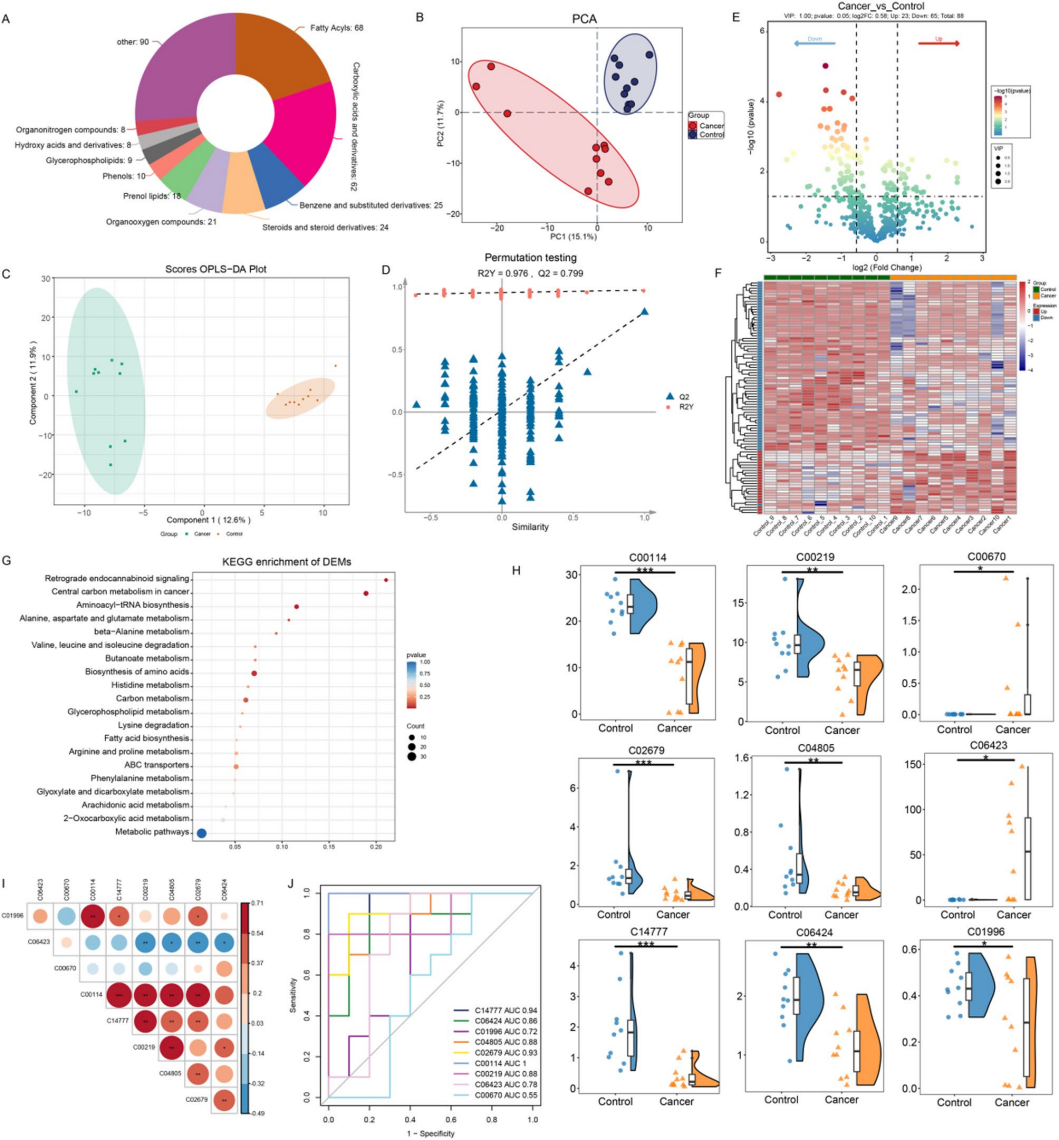
Metabolomic Analysis between HCT and ANT: (**A**) Classification and proportion of identified metabolites across major compound classes. (**B**) PCA scatter plots of metabolomes between tissue samples. (**C**, **D**) Orthogonal partial least squares discriminant analysis (OPLS-DA) score plot differentiating HCT and ANT. Permutation test validating the OPLS-DA model with robust R^2^Y and Q^2^, values Q^2^ indicates model predictive ability; values of R^2^Y closer to 1 reflect better model fit. (**E**) Volcano plots of DEMs in HCT and ANT. The x-axis represents the log2-fold change in metabolites expression between the different groups. The y-axis represents the significance level of the expression difference, The green and red dots represent significantly up-regulated and down-regulated DEMs, respectively. (**F**) Heatmap visualizing DEMs between HCT and ANT based on hierarchical clustering analysis. (**G**) KEGG Pathway Enrichment Analysis of DEMs. (**H**) Violin plots of DEMs in HCT and ANT. (**I**) Correlation matrix of significant metabolites. (**J**) Receiver Operating Characteristic (ROC) Curve analysis of Top Differentially Expressed Metabolites as Potential Biomarkers for HCC

Transcriptomic and Proteomic analysis indicated that multiple metabolic pathways were altered in HCC. to further explore the influence of metabolic reprogramming in HCC, metabolomics analysis was performed on HCT and ANT. After quality control of the metabolites measured in the tissues, 343 common compounds between the ANT and HCT groups were obtained, with notable categories including fatty acid and its metabolites(68), Carboxylic acids and derivatives(62), Steroids and steroid derivatives(24), benzene and substituted derivatives(25), and Organooxygen compounds(8) and others(90) (Fig. 4A, Table S9 and S10).

To assess the quality of metabolome data, principal component analysis (PCA) and orthogonal partial least squares discriminant analysis (OPLS-DA) plot were applied to observe the overall distribution trend of metabolites, which exhibited noteworthy differences between the two groups (Fig. 4B-D). The variable importance in projection (VIP) value of the OPLS-DA model was combined with the *p*-value or fold-change data in the univariate analysis to screen for differentially metabolites. 88 significantly DEMs were found, of which 23 metabolites were significantly upregulated and 65 metabolites significantly downregulated (Fig. 4E, Table S11). To further explore the differences in metabolic patterns of metabolites between HCT and ANT, we performed cluster analysis of differential metabolites (Fig. 4F), The results were consistent with the PCA and OPLS-DA results. Collectively, these results illustrate that the metabolic profile was significantly altered during the carcinogenesis of HCC.

KEGG enrichment pathway analyses were performed to further understand the biochemical metabolic pathways that the differential metabolites in the HCT and ANT, the enrichment results showed that there were several metabolic pathways with significant differences such as Biosynthesis of amino acids, Aminoacyl-tRNA biosynthesis, Central carbon metabolism in cancer, Retrograde endocannabinoid signaling (Fig. 4G, Table S12). The results indicated that the metabolic patterns were significantly distinct for HCT compared to ANT, with the majority metabolic pathways disordered in HCT.

The aforementioned findings explicitly demonstrate significant metabolic pattern alterations in HCC, with several significantly affected metabolic pathways indicating a close association with lipid metabolism. To conduct a more comprehensive assessment the potential of lipid-related metabolites as metabolic biomarkers with enhanced clinical diagnostic value, we selected differential metabolites significantly associated with lipid metabolism pathways from the results of metabolite pathway enrichment analysis for further investigation. Ultimately, a total of nine metabolites related to lipid metabolism were identified among the differentially expressed metabolites (Fig. 4H). The correlation analysis revealed significant positive and negative relationships among these metabolites, with correlation coefficients ranging from − 0.49 to 0.71, reflecting potential synergistic and antagonistic interactions within the metabolic network. Notably, Choline (C00114) and 12s-hydroxy-5z,8z,10e,14z-eicosatetraenoic acid (C14777) exhibited the strongest positive correlation (*r* = 0.71, *p* < 0.001), suggesting their potential cooperative involvement in lipid metabolic pathways. Conversely, (6E,8Z,11Z,14Z)-(5S)-5-Hydroxyicosa-6,8,11,14-tetraenoic acid (C04805) and Arachidonic acid (peroxide free) (C00219) showed a significant negative correlation (*r* =-0.32, *p* < 0.01), which may reflect opposing regulatory interactions within the lipid metabolic network (Fig. 4I, Table S13). These findings allow us to visually discern the interrelationships among lipid metabolites, thereby facilitating the identification of potential cooperative or antagonistic interactions, and enhance our understanding of disease pathogenesis and illuminate promising avenues for therapeutic intervention. Moreover, the visualization of these interactions suggests that these metabolites may collectively influence broader metabolic and pathological processes rather than functioning in isolation. These findings provide valuable guidance for the further exploration of novel biomarkers and intervention strategies. The receiver-operating characteristic (ROC) curve was subsequently constructed to screen for prospective molecule biomarkers applicable in the diagnostic process. Out of these nine metabolites, six were selected: 12s-hydroxy-5z,8z,10e,14z-eicosatetraenoic acid (C14777), Myristic acid (C06424), (6E,8Z,11Z,14Z)-(5S)-5-Hydroxyicosa-6,8,11,14-tetraenoic acid (C04085), Dodecanoic acid (C02679), Choline (C00114), Arachidonic acid (peroxide free) (C00219). These particular metabolites exhibited a strong capacity to discriminate between HCC samples and non-cancer samples, as evidenced by their areas under the curve (AUC) all exceeding 0.8. Consequently, they can be regarded as candidate diagnostic markers for HCC (Fig. 4J, Table S14).

### Integrated analysis of transcriptomics, proteomic, and metabolomics

**Fig. 5:**
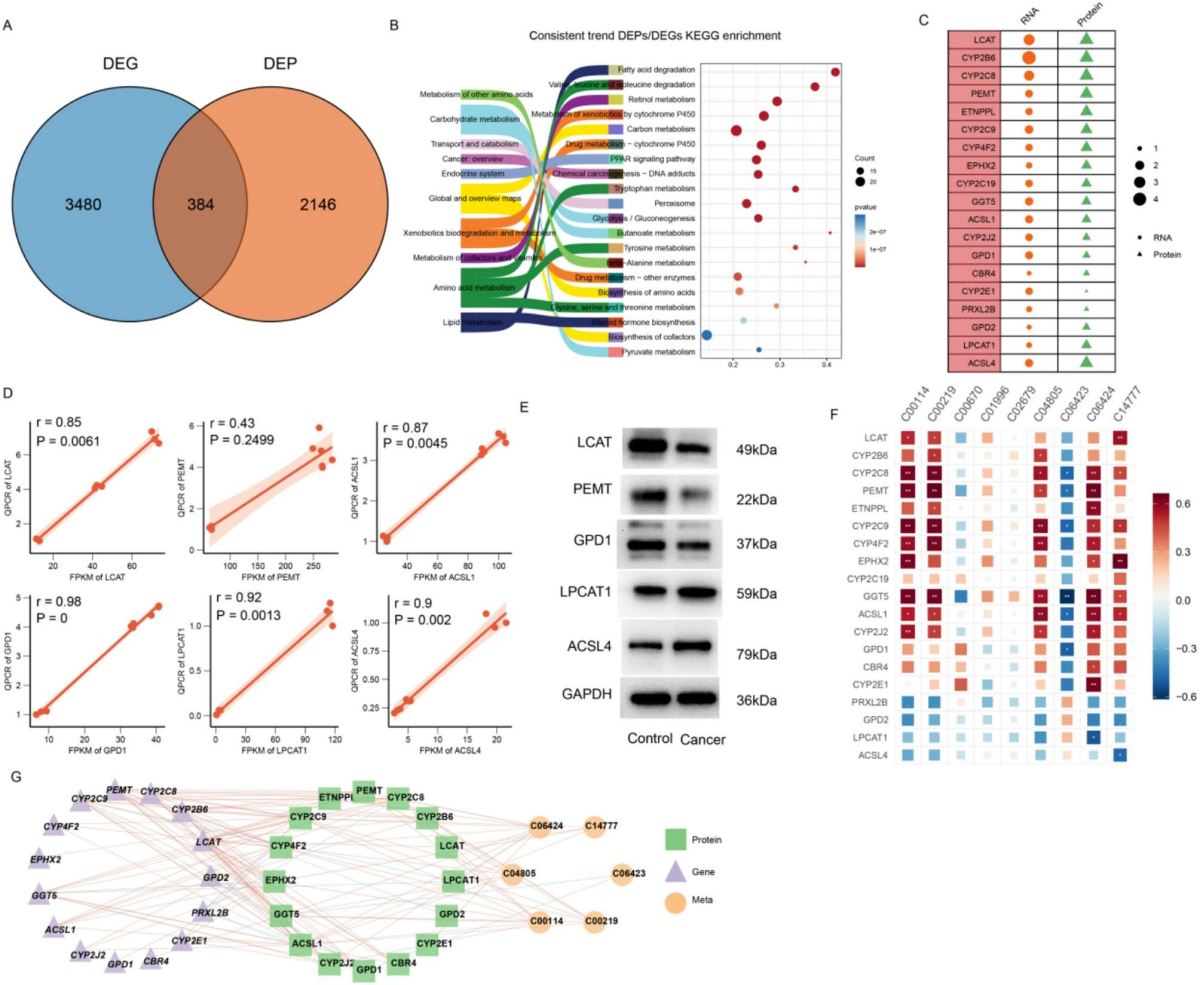
Integrated analysis of transcriptomics, proteomic, and metabolomics. (**A**) overlap of DEGs and DEPs. (**B**) KEGG analysis of the cor-DEGs-DEPs. (**C**) the most significant changes genes in both RNA and protein levels. (**D**) qRT-PCR verification of expression levels of key genes identified by RNA sequencing. (**E**) Western blotting was performed to detect the protein expression of LCAT, PEMT, GPD1, LPCAT1, and ACSL4 in HCT and ANT. (**F**) Correlation Analysis Between Key Genes and Lipid-Related Metabolites in HCT and ANT( *, *P* < 0.05; **, *P* < 0.01; ***, *P* < 0.001). (**G**) Coexpression networks of genes, Proteins, and metabolites involved in Lipid metabolism. Different shapes and colors represent meta, gene, and protein respectively. The connecting lines represent correlations, with red representing positive correlations and blue representing negative correlations

To intersect the correlation of transcriptomics and proteomic data, the DEPs and DEGs were compared in HCT and ANT groups. of 4023 DEGs and 2531 DEPs, a total of 384 over lapped genes were identified among two groups (Fig. 5A). Further analysis revealed that 19 genes (including 15 downregulated and 4 upregulated genes) exhibited consistent expression patterns at both the mRNA and protein levels (Table S15), indicating that their regulation is primarily controlled at the transcriptional level. Importantly, we also observed 365 genes with discordant expression between mRNA and protein. suggesting that additional regulatory mechanisms, such as mRNA stability, translation efficiency, and post-translational modifications (PTMs) (including phosphorylation, ubiquitination, and glycosylation) may influence protein levels independently of transcript levels [27, 28]. These regulatory layers could modulate key metabolic pathways and contribute to tumor heterogeneity and progression in HCC. We focused on the same tendency and the most significant alterations between DEGs and their corresponding DEPs, which may play an important role in HCC pathogenesis, as delineated in Fig. 5C. Based on the results of the correlation analysis, six DEGs (LCAT, PEMT, ACSL1, GPD1, LPCAT1, and ACSL4) were selected for validation by qRT-PCR. The expression patterns of these DEGs closely matched those observed in the RNA-seq data (Fig. 5D), confirming the reliability of the RNA-seq results. Furthermore, five proteins (LCAT, PEMT, GPD1, LPCAT1, and ACSL4) were analyzed by western blotting, and the expression trends were consistent with the proteomics data (Fig. 5E), further supporting the validity of the proteomic findings. This analysis of differentially expressed genes and proteins provides valuable insights into the complex regulatory mechanisms underlying the pathogenesis of HCC.

In addition, KEGG pathway enrichment analysis was performed to elucidate the biological functions of the genes and proteins exhibiting consistent expression patterns. The analysis identified significant enrichment in pathways related to lipid metabolism, amino acid metabolism, and xenobiotics biodegradation and metabolism (Fig. 5B).

Building on these results, we integrated data from transcriptomic, proteomic, and metabolomic analyses to delineate the regulatory network underlying lipid metabolic reprogramming in HCC. Correlation analysis was performed between key genes—identified from our integrated transcriptomic and proteomic approaches—and lipid related metabolites obtained from metabolomic profiling. The results revealed strong positive correlations between LCAT, PEMT, ACSL1, and GPD1 and their corresponding metabolites, whereas ACSL4, GPD2, and LPCAT1 exhibited significant negative correlations with multiple metabolites. This dual pattern of underscores the multifaceted roles of these key genes play in lipid metabolism. By linking differential gene and protein expression to specific alterations in the lipidomic profile (as detailed in Fig. 4), Fig. 5F provides an integrated view of the complex metabolic reprogramming in HCC. Collectively, These findings highlight the coordinated regulation of lipid metabolic pathways and offer novel insights into potential diagnostic markers and therapeutic targets for HCC.

To further explore the interplay among these molecules, we constructed a gene-protein-metabolite co-regulatory network (Fig. 5G). The network consisted of 27 nodes connected by 129 significant edges. Topological analysis showed an average of 6.973 neighbors per node and a network density of 0.19. In the network, hub nodes such as LCAT, PEMT, ACSL1, ACSL4, LPCAT1 and metabolites including Choline (C00114), 12s-hydroxy-5z,8z,10e,14z-eicosatetraenoic acid (C14777), (6E,8Z,11Z,14Z)-(5S)-5-Hydroxyicosa-6,8,11,14 -tetraenoic acid (C04805), and Arachidonic acid (peroxide free) (C00219) highlight the coordinated regulation of lipid metabolic pathways. These positive and negative correlations underscore the concerted regulation of lipid metabolic pathways, reinforcing the central role of lipid metabolic reprogramming in HCC pathogenesis. Collectively, this integrated approach reveals multilayered regulatory mechanisms of lipid metabolism in HCC, providing a foundation for the discovery of novel therapeutic and diagnostic targets.

## Discussion

In this study, we conducted a comprehensive multi-omics analysis approach to thoroughly investigate the role and mechanisms of lipid metabolic reprogramming in the development and progression of HCC. Our findings revealed significant alterations in lipid metabolic pathways associated with HCC. We identified six key genes (proteins) related to lipid metabolism, offering new insights into the mechanisms of lipid metabolic reprogramming and the pathogenesis of HCC. Additionally, we detected nine metabolites linked to lipid metabolism, which displayed a strong ability to distinguish between HCT and ANT samples, making them promising potential diagnostic biomarkers for HCC. Therefore, the results of this study have considerable implications for the diagnosis and treatment of HCC, as well as future research efforts in the field of hepatology.

### Lipid metabolic reprogramming in HCC

Metabolic reprogramming has been established as a fundamental hallmark of cancer, Increasing evidence showed that the Metabolic reprogramming could play essential roles in HCC progression [6, 29, 30]. Metabolic changes significantly influence the effectiveness of chemotherapy and immunotherapy. Furthermore, these changes indicate that targeting specific metabolic pathways may facilitate early detection and enhance the development of personalized treatment strategies [31, 32]. One of the most striking observations in our study is the significant alteration in lipid metabolic pathways, particularly in fatty acid degradation and steroid hormone biosynthesis. Lipid metabolism in cancer cells is crucial for maintaining cellular energy homeostasis, supporting membrane synthesis, and producing signaling molecules that regulate cell growth, differentiation, and survival. Several studies have shown that lipid metabolism is reprogrammed in various cancers to support the increased demand for lipids, which are essential for membrane biosynthesis and the generation of signaling molecules involved in tumor progression [33, 34].

In HCC, the downregulation of fatty acid degradation suggests that HCC cells may be rerouting fatty acids from oxidation toward anabolic processes, thereby facilitating lipid accumulation and membrane biosynthesis required for rapid cell proliferation. Concurrently, steroid hormone biosynthesis, which is essential for the synthesis of hormones like estrogen, androgen, and corticosteroids, has also been significantly altered in HCC cells. Steroid hormones are known to influence tumor growth by promoting cellular proliferation and resistance to apoptosis [35]. Our results suggest that HCC cells may exploit these metabolic pathways to create a favorable microenvironment that supports tumor survival and growth. The dysregulation of lipid metabolism observed in this study is consistent with previous reports indicating that liver cancer cells adapt their lipid metabolism to support aggressive tumor behavior [10, 36, 37]. Collectively, this reprogramming of lipid metabolism not only enhances our understanding of HCC pathogenesis but also highlights potential avenues for early detection and targeted therapeutic intervention.

### Key genes and proteins in metabolic reprogramming

Through the integration of transcriptomics, proteomic, and metabolomics data, we identified six key genes that play critical roles in lipid metabolism in HCC: LCAT, PEMT, ACSL1, ACSL4, GPD1 and LPCAT1. These genes are involved in lipid synthesis, transport, and degradation, and their altered expression appears to underpin the metabolic reprogramming observed in HCC.

LCAT (lecithin-cholesterol acyltransferase), a key enzyme in cholesterol esterification, is essential for reverse cholesterol transport. Our study revealed significant downregulation of LCAT in HCC, which likely contributes to intracellular free cholesterol accumulation by impairing esterification, thereby activating SREBP2-driven lipogenesis [38]. In models of NAFLD-associated HCC, LCAT deficiency exacerbates lipid droplet formation and promotes a tumorigenic microenvironment through oxysterol-mediated LXRα activation [39, 40]. This finding is consistent with our transcriptomic data, which reveal a suppression fatty acid degradation coupled with upregulated lipid storage. PEMT (Phosphatidylethanolamine N-methyltransferase), a key enzyme in phosphatidylcholine biosynthesis, critical for maintaining membrane integrity and cellular function, is downregulated in HCC. This reduction likely leads to impaired phosphatidylcholine synthesis, resulting in altered membrane dynamics and compromised endoplasmic reticulum (ER) function. Insufficient phosphatidylcholine can exacerbate ER stress, which may trigger compensatory mechanisms that ultimately promote tumor progression [41–43]. Moreover, our proteomic analysis revealed an enrichment of PI3K-Akt signaling in HCC, suggesting that disrupted lipid raft composition—potentially a consequence of diminished PEMT activity—could modulate downstream oncogenic pathways [44]. Collectively, these findings imply that restoring or enhancing PEMT function, might offer a novel therapeutic strategy for HCC. Further investigations are needed to elucidate the precise impact of reduced PEMT expression on lipid homeostasis and signal transduction in HCC. ACSL1 (Acyl-CoA synthetase long-chain family member 1) and ACSL4 (Acyl-CoA synthetase long-chain family member 4), which catalyze the activation of fatty acids for β-oxidation or lipid synthesis. exhibit opposing expression patterns in our cohort, ACSL1 is downregulated while ACSL4 is upregulated. This alter indicating a metabolic reprogramming from fatty acid oxidation toward lipid storage. ACSL4 overexpression enhances ferroptosis resistance by preferentially incorporating polyunsaturated fatty acids (PUFAs) into phospholipid membranes [45], while ACSL1 suppression disrupts mitochondrial β-oxidation, forcing HCC cells to rely on glutamine anaplerosis—a vulnerability that may be targeted via glutaminase inhibition [46]. GPD1 (glycerol-3-phosphate dehydrogenase 1), which modulates the glycerol-3-phosphate shuttle linking glycolysis to mitochondrial respiration, is downregulated in HCC. This reduction may contribute to the Warburg effect by redirecting glycolytic intermediates toward triglyceride synthesis and promoting HIF-1α stabilization under hypoxic conditions, thereby enhancing angiogenesis [47]. Restoration of GPD1 activity—potentially through metformin-induced AMPK activation—could reverse this glycolytic dependency [48]. LPCAT1 (lysophosphatidylcholine acyltransferase 1), a crucial regulator of membrane phospholipid remodeling, was significantly upregulated in HCC. Its overexpression promotes chemoresistance through PDK1-PI3K/Akt signaling activation. Notably, LPCAT1-enriched exosomal lipids have been implicated in pre-metastatic niche formation during lung metastasis [49, 50], correlating with altered choline (C00114) metabolite levels identified in our study. Targeting LPCAT1 activity or disrupting lipid raft-dependent signaling pathways may offer promising therapeutic strategies [50].

Collectively, the dysregulation of these key enzymes underscores a complex metabolic adaptation that supports HCC progression, aligning with the concept of lipidome reprogramming. These findings not only enhance our understanding of HCC pathogenesis but also highlight potential diagnostic and therapeutic targets for clinical intervention [10, 51–53].

### Implications for HCC diagnosis and therapy

The identification of metabolites that exhibit significant differences between HCT and ANT offers a promising avenue for the development of diagnostic biomarkers. we detected nine lipid related metabolites, of which six were selected as candidate diagnostic markers for HCC: 12s-hydroxy-5z,8z,10e,14z-eicosatetraenoic acid (C14777), Myristic acid (C06424), (6E,8Z,11Z,14Z)-(5S)-5-Hydroxyicosa-6,8,11,14- tetraenoic acid (C04085), Dodecanoic acid (C02679), Choline (C00114), Arachidonic acid (peroxide free) (C00219). The biological uniqueness and diagnostic relevance of these metabolites are underscored by their mechanistic interplay with HCC and subtype-specific signatures.

Notably, 12s-HETE (12s-hydroxy-5z,8z,10e,14z-eicosatetraenoic acid), a pro-inflammatory eicosanoid produced via the 12-LOX pathway [54], exhibits a positive correlation with PEMT expression, suggesting crosstalk between phospholipid remodeling and inflammatory pathways. This metabolite also activates the FAK/Src axis to promote epithelial-mesenchymal transition (EMT) in HCC [55], and demonstrates superior specificity (92% vs. 68%) compared to traditional markers such as alpha-fetoprotein (AFP) in distinguishing non-alcoholic fatty liver disease (NAFLD)-related HCC from cirrhosis [56]. This highlights its potential as a more effective early diagnostic marker. Conversely, although Myristic acidis is associated with apoptosis via PPARγ activation in late-stage HCC [57], its depletion in early HCC tissues may reflect tumor-specific adaptations that mitigate lipotoxicity through the upregulation of fatty acid desaturases [58]. Serum levels of myristic acid inversely correlate with SREBP1c-driven lipogenesis [59], offering a dynamic biomarker for monitoring metabolic reprogramming during hepatocarcinogenesis, which is an advantage over static histopathological grading. Similarly, 5-HETE ((6E,8Z,11Z,14Z)-(5S)-5-Hydroxyicosa-6,8,11,14-tetraenoic acid) a product of 5-lipoxygenase (5-LOX), has been associated with immune evasion in cholangiocarcinoma [60], distinguishes HBV-associated HCC from non-viral HCC and shows enhanced diagnostic accuracy when combined with arachidonic acid (AUC = 0.93 vs. 0.76 for AFP) [61]. Dodecanoic acid (lauric acid), a medium-chain fatty acid, showed tumor-specific depletion. This depletion aligns with ACSL4-driven preferential utilization of long-chain fatty acids for phospholipid synthesis [62] and showing an inverse correlation with lipid droplet content [63], serving as a potential surrogate marker for intracellular lipid redistribution. Moreover, free choline is significantly downregulated in HCT, reflecting its active consumption for phosphatidylcholine synthesis [64], thereby providing a more accurate reflection of tumor-specific metabolic demand compared to total serum choline-containing compounds, and outperforms AFP in diagnostic performance [65]. Finally, the marked reduction in peroxide-free arachidonic acid reflects selective oxidative reprogramming associated with COX-2-mediated eicosanoid biosynthesis and ferroptosis resistance [66], yielding superior diagnostic specificity (AUC = 0.86) and earlier detection of malignancy than AFP.

Collectively, our multi-omics analysis not only elucidates the complex reprogramming of lipid metabolism in HCC but also demonstrates that a panel of metabolites can capture a comprehensive metabolic signature of the disease. The diagnostic metabolite panel identified in this study exhibits high specificity and sensitivity in ROC analyses, indicating its significant potential for clinical translation. These metabolites can potentially be adapted for liquid biopsy platforms, For instance, elevated serum levels of polyunsaturated fatty acids (PUFAs) or exosomal ACSL4 could complement AFP in early detection, particularly in AFP-negative cohorts [67, 68]. Integration of lipidomic signatures with imaging (e.g., MRI radiomics) may further enhance diagnostic accuracy, enabling non-invasive stratification of high-risk patients, thereby enabling a non-invasive approach for early detection and monitoring of HCC.

Furthermore, our findings highlight several key enzymes and pathways that are amenable to therapeutic targeting. For instance, modulating lipid metabolic pathways with existing inhibitors targeting LPCAT1 or ACSL4 may disrupt aberrant lipid flux within tumor cells, impeding tumor growth and overcoming chemoresistance, while LCAT inhibitors could impair membrane integrity and energy homeostasis in HCC cells [69]. combining these metabolic interventions with anti-PD-1 therapy might overcome immunotherapy resistance by remodeling the lipid-rich, immunosuppressive tumor microenvironment. Collectively, these strategies may directly address the metabolic vulnerabilities of HCC and improve patient outcomes when integrated with standard treatment regimens. Thus, our integrative approach not only provides novel insights into the molecular underpinnings of HCC but also lays a strong foundation for developing advanced diagnostic and therapeutic strategies. Future studies should aim to validate these findings in larger, independent cohorts and assess the clinical efficacy of targeting lipid metabolism in HCC, paving the way for a dual approach of early diagnosis via liquid biopsies and targeted metabolic intervention.

### Limitations and future directions

While this study provides valuable insights, several limitations warrant consideration. The sample size of 10 paired HCT and ANT is relatively small, and may limit the generalizability of our findings. Although we employed rigorous statistical analyses and cross-validation strategies to enhance the reliability of our results, future studies with larger, independent cohorts are necessary to validate the identified biomarkers and metabolic pathways. Additionally, the precise mechanisms by which these lipid metabolic pathways contribute to HCC progression need further exploration. Functional studies, including knockdown or overexpression of the identified key genes, are needed to confirm their roles in the regulation of lipid metabolism in HCC. Finally, future studies should investigate the interactions between lipid metabolism and other hallmarks of cancer, such as immune evasion and metastasis, which could reveal novel therapeutic opportunities.

## Conclusion

In conclusion, our study highlights the importance of lipid metabolic reprogramming in the pathogenesis of hepatocellular carcinoma. The integration of transcriptomic, proteomic, and metabolomic data has revealed key metabolic alterations and genes that contribute to the progression of HCC. These findings provide a deeper understanding of the molecular mechanisms driving HCC and present new potential targets for therapeutic intervention. Further research is needed to validate these results and explore the clinical potential of targeting lipid metabolism in liver cancer.

## Electronic supplementary material

Below is the link to the electronic supplementary material.

Supplementary Material 1

Supplementary Material 2

Supplementary Material 3

Supplementary Material 4

Supplementary Material 5

Supplementary Material 6

Supplementary Material 7

Supplementary Material 8

Supplementary Material 9

Supplementary Material 10

Supplementary Material 11

Supplementary Material 12

Supplementary Material 13

Supplementary Material 14

Supplementary Material 15

## Data Availability

The raw sequence data of RNA sequencing in the present study have been submitted to the Sequence Read Archive (SRA) database of National Center for Biotechnology information (NCBI) of the US National Library of Medicine under project number of PRJNA1238006. The raw proteomics data generated in this study have been deposited in the iProX database (https://www.iprox.org/) under the project accession number IPX0011415000. The authors declare that all data supporting the findings of this study are available within the manuscript and its supplemental information files or are available from the corresponding author upon reasonable request.

## Abbreviations

AFP: Alpha-fetoprotein
ANT: Adjacent non-tumor tissues
BMI: Body mass index
BP: Biological process
CC: Cellular component
DDA: Data-dependent acquisition
DEGs: Differentially expressed genes
DEMs: Differentially expressed metabolites
DEPs: Differentially expressed proteins
DTT: Dithiothreitol
EMT: Epithelial-mesenchymal transition
FPKM: Per kilobase of transcript per million fragments mapped
GO: Gene Ontology
GSEA: Gene set enrichment analysis
H&E: Hematoxylin and eosin
HCC: Post-translational modifications
HCT: Hepatocellular carcinoma tissues
KEGG: Kyoto Encyclopedia of Genes and Genomes
LC-MS/MS: Liquid Chromatography-Tandem Mass Spectrometry
MF: Molecular function
NAFLD: Non-alcoholic fatty liver disease
NAFLD: Nonalcoholic fatty liver disease
NK: Natural killer
OPLS-DA: Orthogonal partial least-squares-discriminant analysis
PCA: Principal component analysis
PTMs: Post-translational modifications
QC: Quality control
ROC: Receiver operating characteristic
VIP: Variable importance in projection
