# Supplementary material for: Metabolic reprogramming in hepatocellular carcinoma: an integrated omics study of lipid pathways and their diagnostic potential
Source: J Transl Med. 2025 Jun 11;23:644. doi: 10.1186/s12967-025-06698-7 (PMC12153152; doi:10.1186/s12967-025-06698-7)
Supplement: Supplementary file 1 — Supplementary Material 1 [file 12967_2025_6698_MOESM1_ESM.docx]

**Supplementary Table S1**

**Summary of Sequencing Data**

| Sample | Raw Reads | Raw Bases | Clean Reads | Clean Bases | Error Rate | Q20 | Total mapped |
| --- | --- | --- | --- | --- | --- | --- | --- |
| C1 | 52492426 | 7.87G | 52350180 | 7.85G | 0.03% | 97.50% | 51888154 (99.12%) |
| C2 | 47773216 | 7.16G | 47642492 | 7.15G | 0.03% | 97.69% | 47093194 (98.85%) |
| C3 | 51057496 | 7.65G | 50953356 | 7.64G | 0.03% | 97.92% | 50450480 (99.01%) |
| C4 | 53591936 | 8.03G | 53460314 | 8.02G | 0.03% | 97.38% | 53052105 (99.24%) |
| C5 | 51608210 | 7.74G | 51493440 | 7.72G | 0.03% | 97.52% | 51061547 (99.16%) |
| C6 | 48989380 | 7.34G | 48910366 | 7.34G | 0.03% | 97.84% | 48541600 (99.25%) |
| C7 | 45118852 | 6.76G | 44968528 | 6.75G | 0.03% | 97.37% | 44539673 (99.05%) |
| C8 | 40746900 | 6.11G | 40452086 | 6.07G | 0.03% | 97.00% | 39760328 (98.29%) |
| C9 | 68871994 | 10.33G | 68654420 | 10.3G | 0.03% | 97.39% | 68001204 (99.05%) |
| C10 | 48342358 | 7.25G | 48072668 | 7.21G | 0.03% | 96.98% | 47168512 (98.12%) |
| N1 | 40916800 | 6.13G | 40693842 | 6.1G | 0.03% | 97.39% | 40109440 (98.56%) |
| N2 | 43779786 | 6.56G | 43524260 | 6.53G | 0.03% | 97.21% | 43083276 (98.99%) |
| N3 | 44092498 | 6.61G | 43941156 | 6.59G | 0.03% | 97.80% | 43482600 (98.96%) |
| N4 | 49548798 | 7.43G | 49409096 | 7.41G | 0.03% | 98.05% | 48970791 (99.11%) |
| N5 | 47992638 | 7.19G | 47738842 | 7.16G | 0.03% | 97.28% | 47245985 (98.97%) |
| N6 | 49474942 | 7.42G | 49188236 | 7.38G | 0.03% | 97.57% | 48646775 (98.9%) |
| N7 | 44993140 | 6.74G | 44768976 | 6.72G | 0.03% | 97.39% | 44320872 (99%) |
| N8 | 43960028 | 6.59G | 43693224 | 6.55G | 0.03% | 97.27% | 43306669 (99.12%) |
| N9 | 43975392 | 6.59G | 43785138 | 6.57G | 0.03% | 97.47% | 43338200 (98.98%) |
| N10 | 45338164 | 6.8G | 45168946 | 6.78G | 0.03% | 97.40% | 44765184 (99.11%) |
